# Reading between the lines: Novel insights on wild Pacific harbour porpoise (*Phocoena phocoena vomerina)* social communication through narrow-band high frequency click trains

**DOI:** 10.1371/journal.pone.0317727

**Published:** 2025-02-12

**Authors:** Amy Migneault, Karina Dracott, Nick Tregenza, Chloe V. Robinson

**Affiliations:** 1 Whales Initiative, Ocean Wise, Vancouver, British Columbia, Canada; 2 Chelonia Limited, Mousehole, Cornwall, United Kingdom; Universidade de Sao Paulo Campus de Sao Paulo: Universidade de Sao Paulo, BRAZIL

## Abstract

For cetaceans that produce narrow-band high-frequency click trains such as the Pacific harbour porpoise (*Phocoena phocoena vomerinae*), social acoustic behavior is poorly understood. While harbour porpoises have a reputation of being generally non-social and often solitary, few studies have aimed to quantify acoustic social communication for this species. In the waters surrounding the Port of Prince Rupert in British Columbia, Canada, harbour porpoises are often seen in groups where they have been observed attempting mating and surface-active behaviors. To assess the extent of social communication amongst porpoises in this region, we analyzed a long-term passive acoustic monitoring F-POD dataset collected from two sites, coupled with a detailed social acoustic criterion for detecting patterns of non-foraging click trains. Based on these criteria, porpoises were found to be producing patterns of obvious, discrete, and repetitive click trains that were marked as social. Generalized additive models were used to identify significant temporal trends in the dataset. On average, 5.3% of click trains produced by porpoises were social. Monthly and diurnal fluctuations in social detection positive minutes (DPM) followed a similar trajectory to non-social DPM, with peak activity observed during periods of darkness and from spring to early summer with a smaller increase in the fall. At one site, 11.1% of the DPM in May were classified as social. In general, proportionally more social DPM were found during periods of more overall DPM, suggesting that porpoises were socially communicating while in proximity to one another. Notably, overall DPM significantly decreased by 53.7% over three years. This novel methodology can be replicated in other regions to gain further insight into the social acoustic behavior of harbour porpoises.

## Introduction

Pacific harbour porpoises (*Phocoena phocoena vomerina*) have been monitored acoustically around the Port of Prince Rupert, located on the northern coast of British Columbia (BC), since 2016 [1]. Harbour porpoises are known to reside in the coastal waters of Prince Rupert year-round, and typically exhibit a combination of nocturnal and seasonal (spring and summer) activity peaks [1]. Diurnal and seasonal trends in echolocation have similarly been reported in other harbour porpoise populations [2–6]. Large aggregations of harbour porpoises (groups of 10 or more individuals) have been visually observed within waters surrounding the Port of Prince Rupert, with upwards of 1,000 individuals documented together during winter months [1]. In this region, mating attempts, surface active behavior, and mother-calf pairs are frequently observed during aggregations. More recently, larger groups of harbour porpoises have also been reported in southern BC, with some reports including observations of surface-active behavior and mating attempts [7].

Despite these reports, the harbour porpoise has historically been considered an elusive, less social, and a more solitary marine species, often found in group sizes of 1–2 individuals in BC waters [8]. However, sightings gathered by the Ocean Wise Sightings Network in the waters surrounding Prince Rupert suggest larger group sizes, averaging of 2–4 individuals [1]. Porpoises produce narrow-band high-frequency (NBHF) clicks, which are thought to be used predominantly for echolocation (i.e., foraging and navigation) potentially limiting their capacity for social communication compared to whistles and broad-band clicks produced by other odontocete species such as delphinids [9]. This, combined with the lack of research on wild populations of harbour porpoise compared to other coastal cetacean species (i.e., dolphins), has resulted in a significant gap in understanding of harbour porpoise social communication.

Other taxa, apart from *Phocoenidae,* that have evolved to produce NBHF clicks include *Kogiidae, Pontoporiidae,* and certain dolphin species from genus *Cephalorhynchus* [10–13]. While it was thought that NBHF clicks were likely limited to echolocation purposes [14,15], Hector’s dolphins (*Cephalorhynchus hectori*) have been observed using NBHF clicks for social communication, particularly in association with aerial and aggressive behaviors [16]. The evolution of NBHF clicks could have been driven by the need to avoid killer whale predation (*Orcinus orca*), as NBHF clicks are undetectable to killer whales [11,17], although evading killer whales is likely not the only influence on NBHF evolution [9]. Delphinids, such as bottlenose dolphins (*Tursiops truncates*), produce trains of high frequency clicks during aggressive interactions [18]. Other species of dolphin such as the white-beaked dolphin (*Lagenorhynchus albirostris*) produce high click rate bursts with rates thought to be too high for echolocating and rather are used for communication among individuals in a group [19]. While evidence exists that for some small odontocetes, social communication may be mediated by high click rate bursts and trains, there remains a notable data gap in research that explores the extent to which vocalizations may be socially important to NBHF species [20].

The acoustic characteristics of harbour porpoise NBHF echolocation click trains during the prey ‘searching’ phase begins slow in click rate (with some variation), increases during the prey ‘approach’ phase, and ends with a high click rate feeding buzz, also known as an ‘upsweep’ in PAM data [21]. Social click trains have been reported to differ from foraging click trains based on the absence of an extended series of inter-click intervals (ICI), exceeding 10 ms, which when foraging, the ICI decreases over time during prey searching and successful capture [20,22,23]. Observations of social communication in harbour porpoises are limited, with most studies conducted on captive and/or trained individuals [14,15]. These studies suggest that porpoises appeared to produce distinct, repetitive, high click rate (250–1,000 clicks/s) social calls separated by intervals of silence, particularly during periods of aggression and/or human intervention, indicating that these calls were distinct from foraging behavior [14,15,24]. Similar results were observed following the handling and tagging of a wild porpoise with a nearby calf, during which these repetitive social click trains (>1,000 clicks/s) were recorded [20]. Non-acoustic studies on wild populations of harbour porpoise have documented social gatherings and behaviors such as mating [7] and potential socially coordinated group hunting [25].

In the current literature, there has been no passive acoustic monitoring (PAM) research conducted on wild harbour porpoise social communication without any direct human intervention or potential influence on normal behavior. In this study, we aimed to 1) identify evidence of acoustic social activity for Pacific harbour porpoise on the northern coast of BC, 2) quantify the approximate time Pacific harbour porpoise spend producing social click trains, and 3) determine temporal trends in acoustic social activity.

## Methods

### Study site and PAM data collection

**Fig 1:**
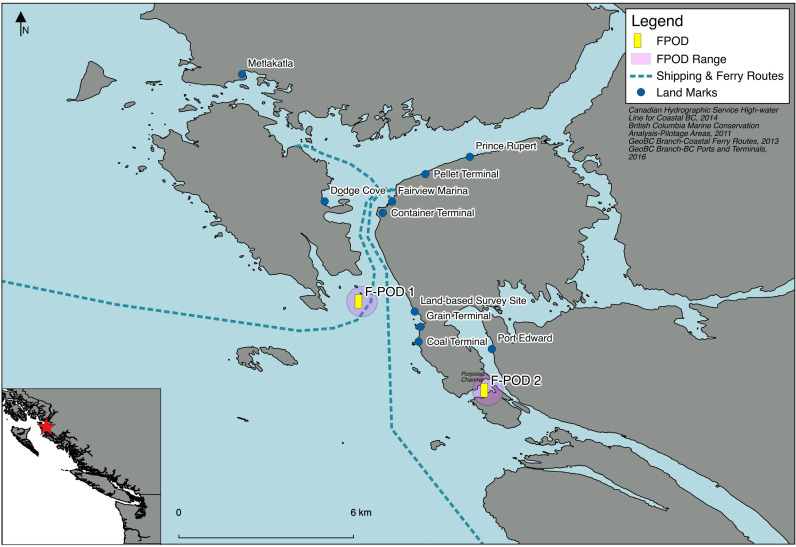
Map depicting F-POD (Full waveform capture – Porpoise Detector) locations in northern British Columbia, Canada.

The primary study site (F-POD 1) consisted of a 2 km^2^ area in Chatham Sound, southeast of Digby Island near Prince Rupert in northwestern BC, Canada [1]. At this site, one PAM device, an F-POD (Full waveform capture – Porpoise Detector) instrument (Chelonia Limited) [26], was deployed between January 24^th^, 2020 - October 3^rd^, 2023 (3.75 years) at a depth of 12 feet from the seabed in 60–80 ft of water, at 54°14’41.9” N 130°21’53.8” W and was intermittently retrieved and immediately replaced by another F-POD at the same location every 3–5 months depending on battery limitations (See [1] for detailed deployment methods). No data was recorded at this site between 8^th^ February 2023 to 4^th^ July 2023 due to a faulty deployment, subsequently resulting in 2023 data being excluded from the site-specific analyses. Our secondary study site (F-POD 2) was located in Porpoise Channel at 54°12’27.5” N 130°17’43.7” W, near the entrance to Port Edward in northwestern BC (Fig 1). An F-POD was moored at the same depth in the southeast side of the channel (approximately 370 m across) and continuously recorded data from 24^th^ March 2022 to 5^th^ June 2023 (1.25 years). The two F-POD locations were 5.9 km apart.

### Social criteria and data processing

After retrieving the F-POD, the files were cropped, and harbour porpoise echolocation click trains were extracted (see details in [1]). Criteria for identifying social calls were developed following recommendations in the literature for distinguishing social click trains based on click rate and repetition [14,15,20]. This was further supported by workshops with Chelonia (detailed software information can be found at https://www.chelonia.co.uk), and detailed social criteria can be found in S1 File. A KERNO-F classifier was applied to identify NBHF harbour porpoise echolocation click trains from the F-POD data [26]. To detect social activity, which typically generates very high rates of NBHF click logging, the F-POD app (F-POD.exe, Chelonia Ltd.) filters were set to show only trains with an average click rate, clicks per second (c/s), of at least 100/s and average click kHz to be between 105–140. Each cropped file that contained at least 256 clicks within 15 second frames were visually analyzed for discrete, high click rate trains (between 200–1,000 c/s). Only groups of click trains that a) were deemed to not be foraging (no visible ‘searching’ phase below 200 c/s or ending in a successful foraging buzz or ‘upsweep’), (b) were not part of a longer train, and (c) demonstrated repeated patterns (at least twice repeated) within a 15 second time frame, and d) ranged between 200–1,000 c/s were marked as social and extracted (S1 File). Social click trains fell predominantly under two categories: “Mushroom” click trains and “Wiggles” (S1 Fig). If there was any indication that the characteristics of the click trains resembled foraging, the train was not marked as social to ensure only obvious social click trains were included in the analysis. For analysis of temporal trends, both “Marked Trains Only” (social trains) and “Marked Trains Excluded” (non-social trains) were exported as detection positive minutes (DPM) per hour and copied into an excel spreadsheet. Non-social trains exclude the marked social trains and are inclusive of all other click trains, such as those used for echolocation.

### Quality control

Each file was analyzed and marked three times using the same method and criteria (S1 File) by the same individual (Individual 1) to ensure no social trains had been missed or falsely marked. To account for potential of subjectivity bias in the visual marking of social trains by Individual 1, a second individual (Individual 2) was given 10% of the unmarked files using random selection and applied the same methodology to independently mark social trains. Data from both individuals were exported and the number of marked social minutes per hour were compared between individuals to determine the level of consistency in marking social trains using this method. Quality control testing revealed that Individual 1 marked 85% of the same social minutes per hour as Individual 2, and Individual 2 marked 93.3% of the same hours as Individual 1. Only the final files marked by Individual 1 were used for the remainder of this study.

### Statistical analysis

#### Proportion of social DPM.

A total of 1,321 days of data between both sites were recorded for analysis between Jan. 24^th^, 2020, and Oct. 3^rd^, 2023 and over 15 million NBHF clicks. Statistical analysis for these data were conducted using R version 4.12 [27] and R Studio version 2021.09.1.372 [28]. To conduct simple descriptive statistics, sites were analyzed separately and the proportion of social DPMs were determined and compared to the total DPM minutes recorded in each hour. Shapiro-Wilk normality tests were used to determine if data was parametric and Kruskal-Wallis testing were used to determine if there were significant differences in the proportion of social DPM across hours, months, and years (between 2020–2022 at Tuck Islet). Post-hoc testing (Dunn’s Test using the Bonferroni correction) was performed following the Kruskal Wallis testing.

#### Generalized additive modelling.

To determine any potential temporal influence on social trains vs non-social trains, Generalized Additive Models (GAM) using the *bam()* and *log link()* functions from R package mgcv [29], were used with negative binomial distribution to account for overdispersion. Social and non-social DPMs were used independently as response variables. Two models per DPM type (social or non-social) were run to test the effects of hour, month, and year separately. Prior to running the models, collinearity was tested using the v*if*() function from the car R package v. 3.0–12 [30] with a cut-off value of 5 [31]. We assessed the response variable for temporal autocorrelation via ACF plots using the *acf*() function (mgcv package), with 0.2 as the threshold [1]. Autocorrelation was detected in our dataset, so subsequent models incorporated an AR1 structure (first-order autoregressive structure with homogenous variances) [32]. Non-significant variables (P >  0.05) were sequentially removed from the model by evaluating the resulting P-values from the Parametric Coefficients and the approximate significance of smooth terms for each variable in the model summary. Final model selection was based on a combination of Akaike’s Information Criterion (AIC) and the adjusted R^2^ values. All plots were created using *ggplot()* function from the R package ggplot2 v.3.3.5. [33].

## Results

### Proportion of social DPM

**Fig 2:**
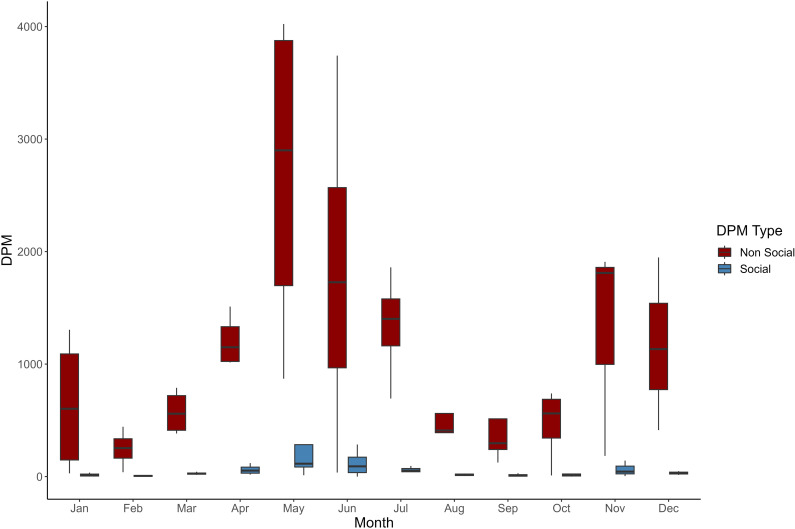
Boxplot of total detection positive minutes (DPM) recorded monthly for both non-social click trains (red) and social click trains (blue).

Within the entire dataset, 2,619 DPM were marked as social out of 49,811 DPM in total. This indicates that porpoises in our study site are producing obvious social calls on average 5.3% of the time they are vocalizing, with variation across months. The proportion of time spent producing social calls differed between the two study sites, Tuck Islet – F-POD 1 (6.7%, or 2,110 DPM) and Porpoise Channel – F-POD 2 (2.6%, or 402 DPM). At Tuck Islet, May was the month with the highest proportion of social DPM at 11.1%, whereas June was the highest month for Porpoise Channel at 6.7%. Kruskal-Wallis tests indicate that differences in the proportion of social DPMs were not significantly influenced by month at either site (Tuck Islet: p-value =  0.7006, Porpoise Channel: p-value =  0.4433) (Fig 2).

**Fig 3:**
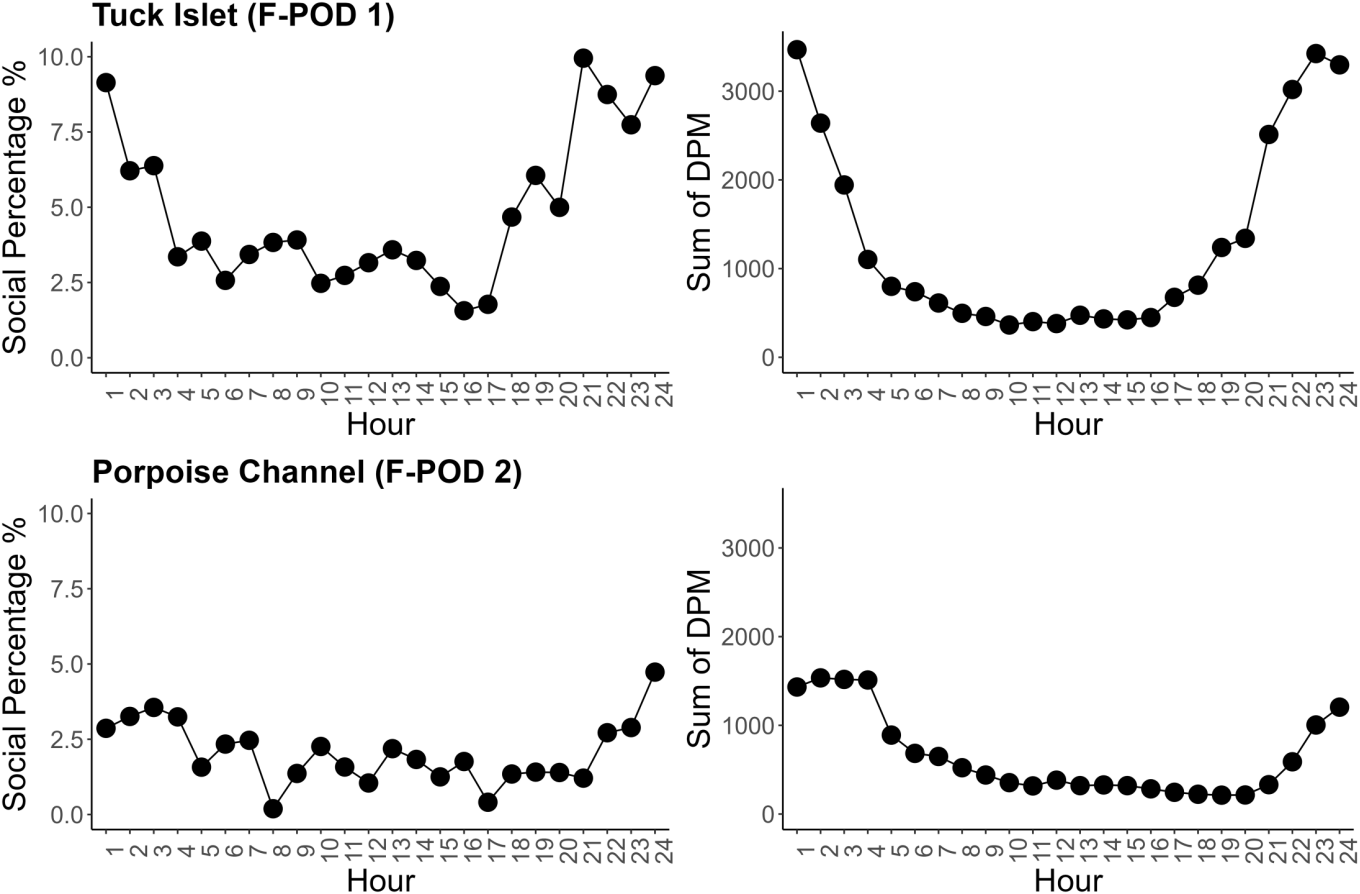
Line chart of the percentage (left) and sum (right) of harbour porpoise social click trains by detection positive minutes (DPM). Top location =  Tuck Islet (F-POD 1), bottom location =  Porpoise Channel (F-POD 2).

At both sites, more social DPM were recorded at night between the hours 21:00 to 3:00. Porpoises spent more time producing social click trains during the hours of darkness, particularly from 21:00–01:00 hr at Tuck Islet. During this time, the percentage of social DPM ranged from 9.1–9.6%. This trend was less apparent at Porpoise Channel, as the highest percentage of social DPM was 4.7% during 24:00 (Fig 3). The Kruskal-Wallis test results indicated that the influence of hour on the proportion of social DPM was not significant.

**Fig 4:**
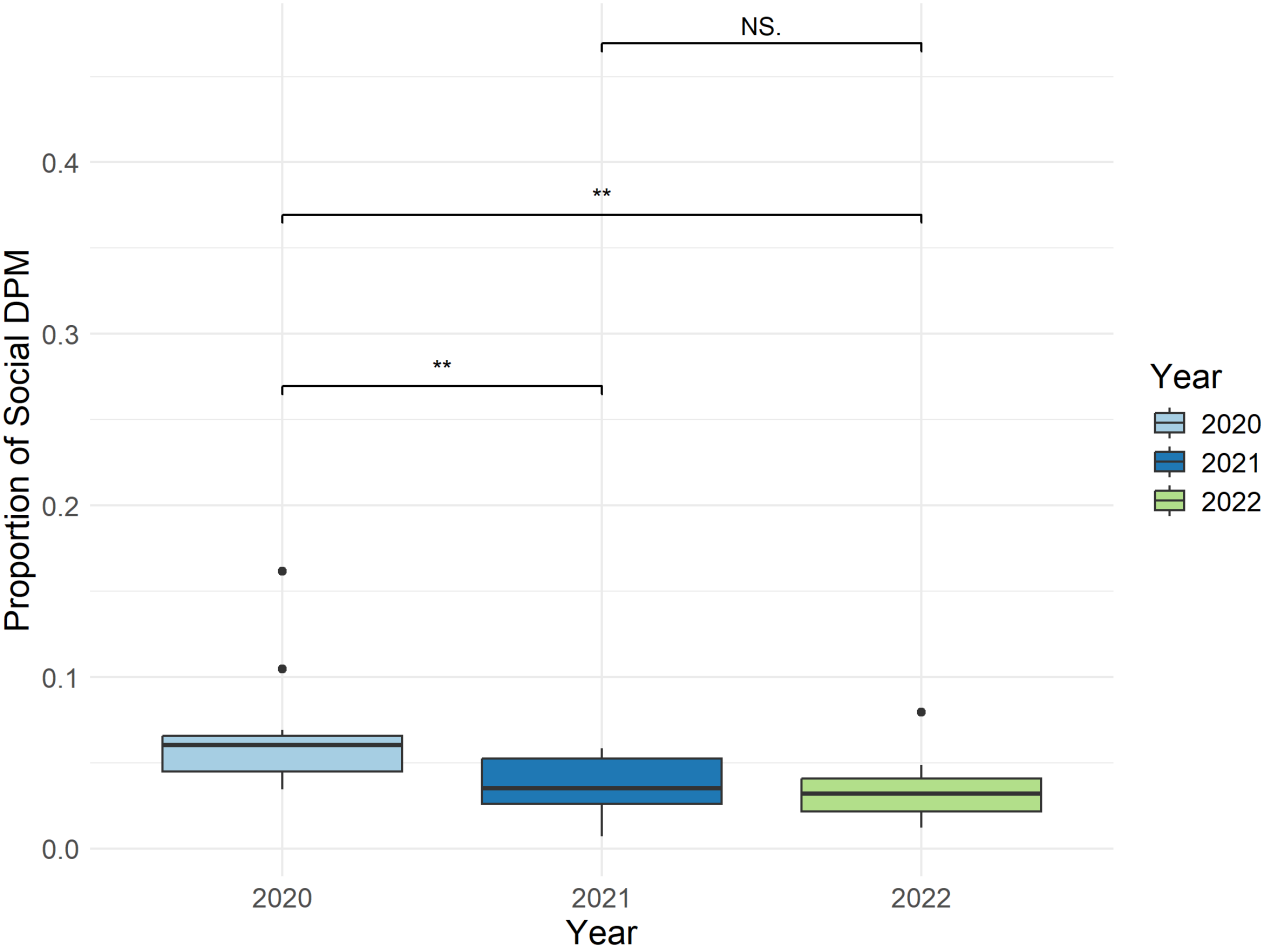
Boxplot comparing proportion of social detection positive minute (DPM) out of overall DPM at Tuck Islet between 2020–2022. ‘**’ indicate significance levels (P >  0.005). ‘NS.’ indicates “Not Significant”.

Annual differences in proportions of social DPM occurred at Tuck Islet. In 2020, 9.3% of DPM were social, and 4.4% in both 2021 and 2022. Overall, the sum of all DPMs (both social and non-social) decreased over time. In 2020, 15,006 DPM were recorded, 9,550 DPM in 2021, and 6,948 DPM in 2022. (S1 Table). Results indicate that the proportion of social DPM compared to total DPM significantly decreased from 2020 to 2022 at Tuck Islet (P <  0.005) (Fig 4).

### Generalized additive models

For Tuck Islet, the final GAM model covariates included sample year (2020–2022), month (January to December), and hour (1–24) with both marked social porpoise DPM and non-social DPM. Results indicate that year, month, and hour all significantly influenced social and non-social DPM, with the model explaining 38.6% and 17% of the variation, respectively. Porpoises displayed greater hourly variability for social click trains compared to non-social click trains at both sites. This was more apparent at Tuck Islet, where social activity by porpoises appears to be more restricted to periods of darkness compared to non-social activity. Social DPM were significantly more prevalent during the months of May (P <  0.001) and June (P < 0.01). Non social DPM also significantly increased in May and July and decreased in September and October (P <  0.05), and February (P <  0.001). Additionally, both social and non-social DPM decreased significantly from 2020 (2021 and 2022: P <  0.001) (S2 Fig).

For Porpoise Channel, the final GAM model included month and hour as covariates but excluded year, with both social and non social DPM. Both month and hour were significant covariates for social DPM. The final model for social DPM explained 27.7% of the variation observed. Both social and non social DPM followed similar hourly trends at Porpoise Channel, with a gradual decrease in DPM between the hours of 1:00 to 17:00, and a sharp increase in DPM following 17:00. There was a significant increase in social DPM during the month of June (P <  0.01). The model for non-social DPM at this site explained 13.8% of the variation observed. During May, June, November, and December non-social DPM were more prevalent and decreased in February (P <  0.01) (S3 Fig).

## Discussion

Typically, it has been assumed that harbour porpoises are solitary, elusive, and less social animals, particularly when compared to other highly social odontocete species [9]. To date, few studies have focused on the social acoustics of harbour porpoise or attempted to quantify this behavior in wild populations [15,20]. The NBHF click trains demonstrated in this study provide novel evidence of diel and seasonal patterns in porpoise communication and may indicate that more social calls are made when porpoise densities are higher. Moreover, this suggests that these click trains are used as communication to conspecifics, and that the vocalizing animal is aware of other porpoises in its surrounding environment, rather than these click trains being used as contact calls to distant porpoises.

Using a long-term PAM data set, this study has identified what are likely discrete social click trains used by Pacific harbour porpoises in northern BC. Marked social trains were identified by high click rate bursts, (200–1,000 clicks/s), with clearly repeated patterns. These click train patterns were notably distinct from any foraging trains ending in an ‘upsweep’ in click rate, characterized by a harbour porpoise tracking a predetermined target [21,24]. When comparing the criteria of a classic foraging click train, the click rate profile of a social click train clearly differs from foraging [20]. However, the ICIs of social click trains overlap with those of feeding click trains. This makes extracting social click trains by ICI difficult for large datasets, especially where multiple porpoises may be vocalizing simultaneously in an area and may be socially communicating in groups during coordinated foraging [25]. These findings highlight the limitations of this study as well as the need to further evaluate how best to distinguish between foraging/navigating echolocation and social communication for harbour porpoise, and successfully extract exclusively social click trains from large datasets. Here, we have identified only the most distinctive forms of social communication criteria for this species, so additional refinement of social communication criteria will result in an increase in the total proportion of time characterized as social communication for harbour porpoise. Visual extraction of social calls can be tedious and subjective, suggesting that incorporating validated automation technology to assist in the extraction of social click trains would expedite the process and decrease subjectivity bias. Developing an automated approach to analyze PAM data would facilitate rapid, large-scale comparison of harbour porpoise social communication and provide further insight into the importance of sociality for this species. Furthermore, studies that incorporate PAM data with land-based, visual surveys could help verify social behavior, group size, and accuracy of automated/AI detection.

From 2020–2022, we found that 5.3% of click trains produced by harbour porpoises were discrete, social click trains. In general, a higher proportion of social click trains were found during periods of greater overall acoustic activity (higher DPM) than during periods of lower acoustic activity (lower DPM). This suggests that porpoises may be socially communicating with one another while in proximity to each other, rather than using these calls for long-distance communication. We also found that porpoises were more social during spring and fall months, and during periods of darkness, which coincided with peak periods of total recorded click trains. These notable seasonal peaks correspond with Chlorophyll *A* concentrations, which can serve as a proxy for presence of prey [1]. This pattern may support the evidence observed of coordinated group hunting, where porpoises are engaging in social communication during hunting tactics to increase the probability of foraging success [25]. This theory aligns with the understanding that social communication has often been thought to be intertwined with foraging and navigating echolocation NBHF click trains [20].

Comparison between sites revealed variation in the proportion of time spent producing social calls, suggesting that harbour porpoises are using the two locations in different ways. Social click trains were more prominent at Tuck Islet, where May was the month with the highest proportion of social DPM at 11.1%, whereas June was the highest month for Porpoise Channel at 6.7%. Social click trains were more restricted diurnally at Tuck Islet compared to Porpoise Channel, where a steadier decrease in social DPM is observed gradually into daylight hours with a sharp increase at dusk. Additionally, there was an increase in activity in Porpoise Channel in November and particularly December compared to Tuck Islet, where there was more activity in spring to summer. These differences may be indicative of temporal shifts in prey availability between the two locations and/or show that porpoises are engaging in different behaviors at different times in these two locations, despite the close proximity between Tuck Islet and Porpoise Channel (3.2 nautical miles). From a spatial perspective, Tuck Islet is a more conducive location for the large aggregations, hunting, and potentially, socially coordinated behaviors, compared to Porpoise Channel, which is a narrow channel. Tuck Islet provides a greater area for such activities which is supported by reported sightings in the region, where larger groups of harbour porpoises have been observed, along with mating attempts, mother calf pairs, and surface-active behavior. Sightings of large groups of harbour porpoise can indicate an influx of prey availability, and/or social gatherings [25,34–36]. These results may indicate the existence of small scale (100 m–6 km) behavioral spatial preferences in this region, both diurnally and seasonally. Notably, harbour porpoise microhabitat preference has been associated with foraging behavior in Sweden, the coast of Washington, and southern BC [6,37,38].

Overall DPM (both social and non-social) decreased by 53.7% from 2020 to 2022 in the Port of Prince Rupert. This is a highly trafficked area by vessels and is one of the fastest growing ports in North America, transporting both cargo and passengers [39], which may suggest vessel disturbance is possible source for this substantial decrease. The port expansion has also resulted in an increase in local industrial construction near both study sites, which includes activities such as pile driving, dredging, and back-filling along shoreline areas [40]. However, there are many factors that could influence this recorded downward trend in activity, including but not limited to masking affects from vessel traffic [41] and other limitations of the F-POD [42]. It should be noted that the F-POD data presented here is generally quiet (average background clicks/minute =  215) and does not show elevated ultrasound levels, but cannot exclude low frequency noise to which porpoises are known to be less sensitive to. There is a known downward bias in detections of porpoises in minutes when other species or sonars are present [26]. This is not a significant factor in this study as the fraction of minutes with either of these biassing factors is < 0.4%. One study in the Baltic Sea noted that there was no decrease in porpoise activity during times of high vessel traffic, but suggested this may be due to an innate need to forage in the study region despite the impact from vessels, and that long-term impacts may still befall the population [43]. Additionally, harbour porpoise presence is often linked to prey availability [4,35,44,45], driven by their high metabolic requirements [46]. In the current study, social DPMs increased significantly in June, which correlates with an increase in overall activity, and likely, an increase in key prey species availability such as surf smelt (*Hypomesus pretiosus*), sand lance (*Ammodyies hexapterus*), and Pacific herring (*Clupea pallasi*) [1,37,47,48]. This may indicate that a decline in porpoise DPM in the Port of Prince Rupert could be related to disturbance and/or reduced prey availability. Moreover, while these factors are likely influencing harbour porpoise acoustic activity (both social and non-social) to some degree, the reason for this decline is difficult to determine without continued research. Furthermore, 2020 marked the beginning of the COVID-19 pandemic, potentially resulting in an abnormal year. Without a baseline established pre-COVID-19, a longer dataset would be required to confirm this downward trend in acoustic activity.

Continued monitoring of harbour porpoise acoustic activity in this study site can help establish important habitat while changes in behavior may indicate shifts in distribution, abundance, or habitat quality. This paper establishes a metric to identify and quantify acoustic social activity in wild harbour porpoises, which can be applied to similar datasets in other regions. This approach allows for broad-scale comparison of social communication and provides further insights into the importance of social communication for this species. Such information is valuable for current and potential future threat mitigation, and for implementing conservation measures to protect at-risk populations of Pacific harbour porpoise.

## Supporting information

S1 File Criteria to Distinguish Harbour Porpoise (Phocoena phocoena) Acoustic Social Behavior from F-POD Files. (PDF)

S1 Table Summary of detection positive minutes (DPM) recorded at Tuck Islet between 2020–2022. (DOCX)

S1 Fig Top panel (A) an example of patterns of harbour porpoise social click trains identified as “Mushrooms”, and bottom panel (B) “Wiggles” recorded using an F-POD in the Port of Prince Rupert. (TIF)

S2 Fig Generalized additive model results for social click trains (top row) and unmarked non-social trains (bottom row) at Tuck Islet from 2020–2022. (TIF)

S3 Fig Generalized additive model results for marked social trains (top) and non-social trains (bottom) at Porpoise Channel. (TIF)
