# Supplementary material for: Reading between the lines: Novel insights on wild Pacific harbour porpoise (Phocoena phocoena vomerina) social communication through narrow-band high frequency click trains
Source: PLoS One. 2025 Feb 12;20(2):e0317727. doi: 10.1371/journal.pone.0317727 (PMC11819512; doi:10.1371/journal.pone.0317727)
Supplement: S1 File — (PDF) [file pone.0317727.s001.pdf]

Criteria to Distinguish Harbour Porpoise (*Phocoena phocoena*) Acoustic Social Behaviour from F-POD Files

<u>F-POD.exe settings (F-POD.exe KERNO-F 1.0, export version 1.04)</u>

- In the “Files” tab, open two FP1 (Amplitude and Clicks/s) and two FP3 (Train Q class and Clicks/s) files.
- In the “Filters + files” tab, filter Clicks/s to be > 100/s in “Train filters”, and filter kHz to be 105-140 in the “Click filters”.
- Using the “Skip to >” function at the bottom left of the screen, skip to > 256 clicks.
- Skip to 256 clicks (bottom left of screen).
- In the left tab, change “Quality” to include: Hi + Mod + Lo.
- Unclick “High sp confidence only”.
- For “Species”, choose “NBHF”, “unclassed”, and DO NOT include “Other cet”.
- Increase y axis of Clicks/s: Hover over clicks/sec file and use **F6** to double scale until “700 Clx/s” is the max number at the top of axis. The default scale is too small to see social trains.

Settings example:

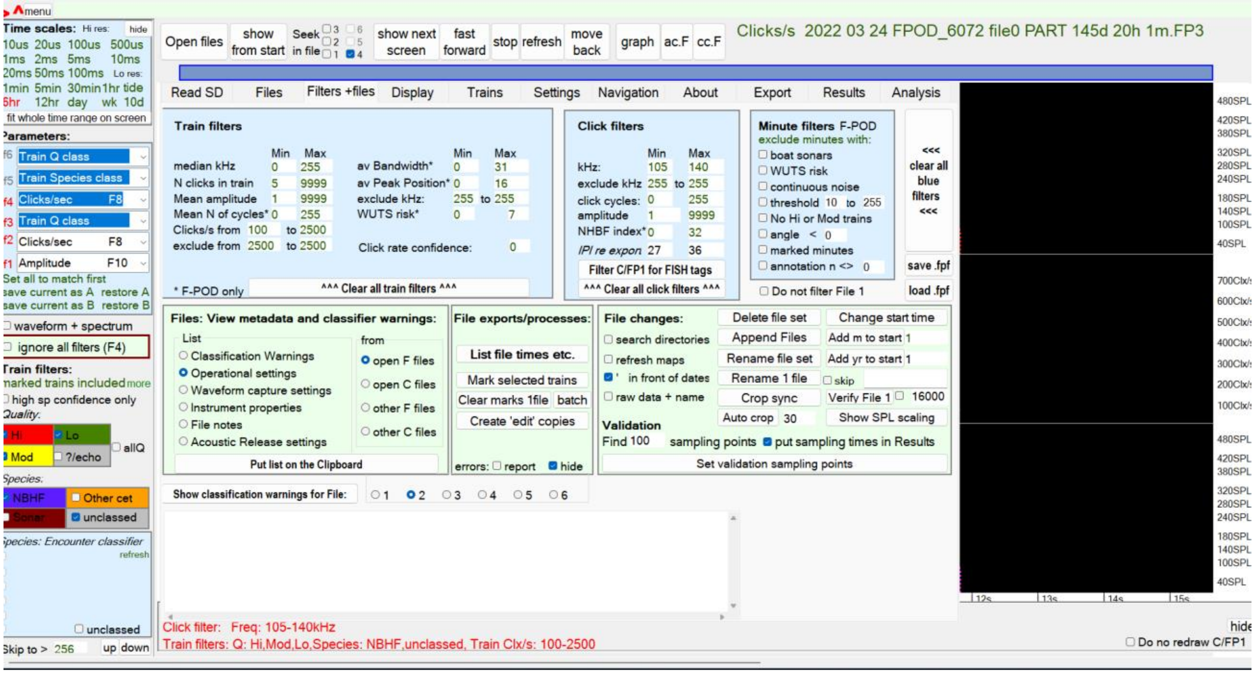

Steps:

1. Start from beginning.
2. Seek in file = 3 or 4.
3. Time frame 15 s (zoom in).
4. Show next screen, focus on clicks/s.
5. Look for **discrete bursts** (could not be apart of a larger train) and **repeated patterns** (at least twice repeated).
    a. **Mushrooms** - Does not have to appear to be a sequence of "**mushrooms**" but needs to not look like foraging (can't start low and build up). A "slow down" at the end is indicative of social communication.

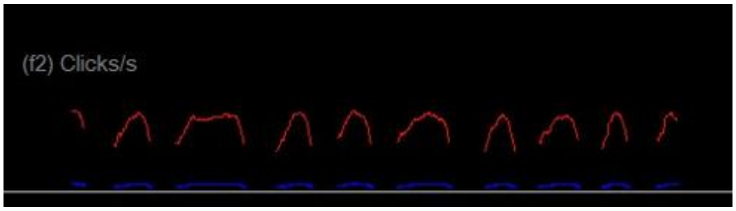

    b. **Wiggling** - If you see a "**wiggling**" or oscillating train, the animal is trying to convey something socially while also navigating/foraging.

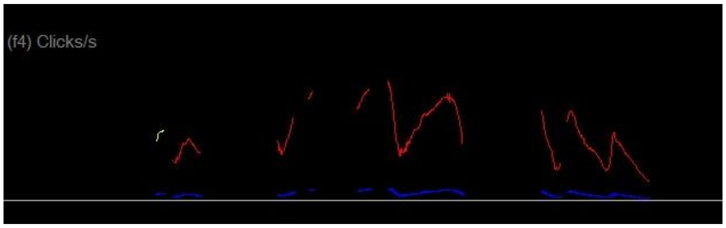

Note: Other patterns, if they occur and are repeated, are possibly identifiable as social: repeated downsweeps as these are not generally seen after the upsweep of a feeding buzz; other 'shapes' that are repeated and appear to be discrete trains.

6. When a screen is of interest and displays clear signs of non-foraging and likely social communication as described above, right click and use "**mark all trains in this minute**" and continue.
7. After a whole file has been gone through and marked, can click under "train filters" until "marked trains only" is selected, and click show from start to examine again and ensure no false markings.
8. If happy with the selected files, go to menu and "Export" then choose the following:
    a. DPM
    b. 1 hour
    c. Minute/day
    d. Results
    e. Export from batch of files

EXAMPLES OF TRAIN MINUTES THAT HAVE BEEN MARKED AS SOCIAL

1.

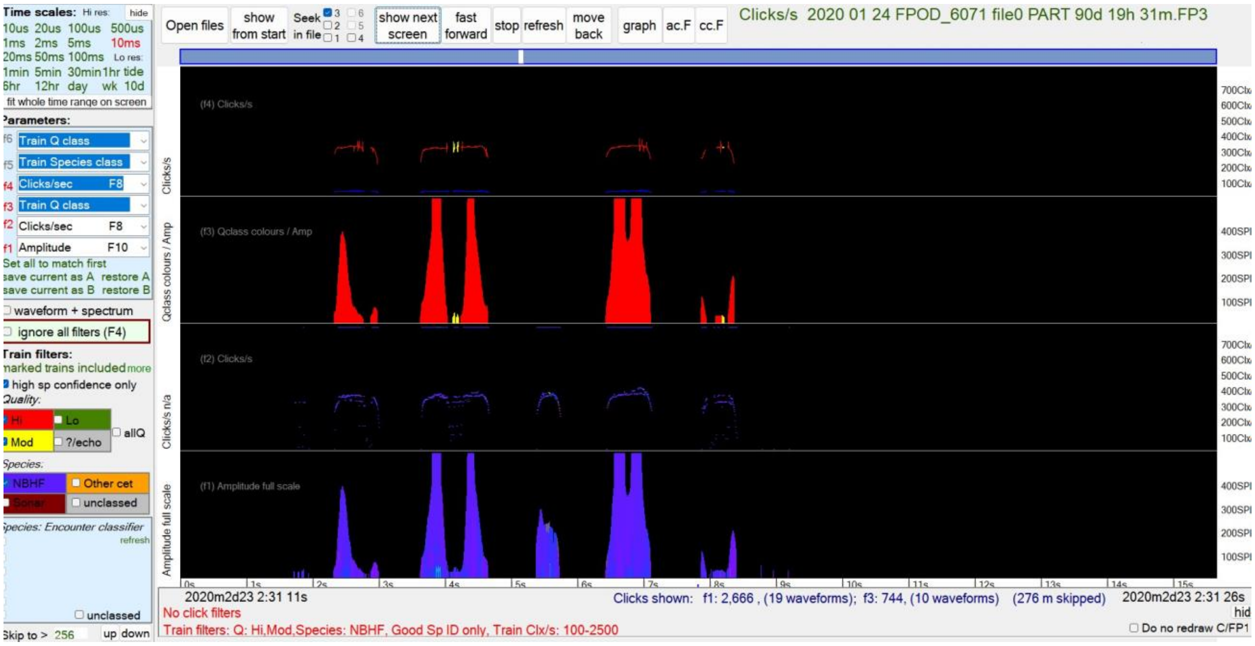

2.

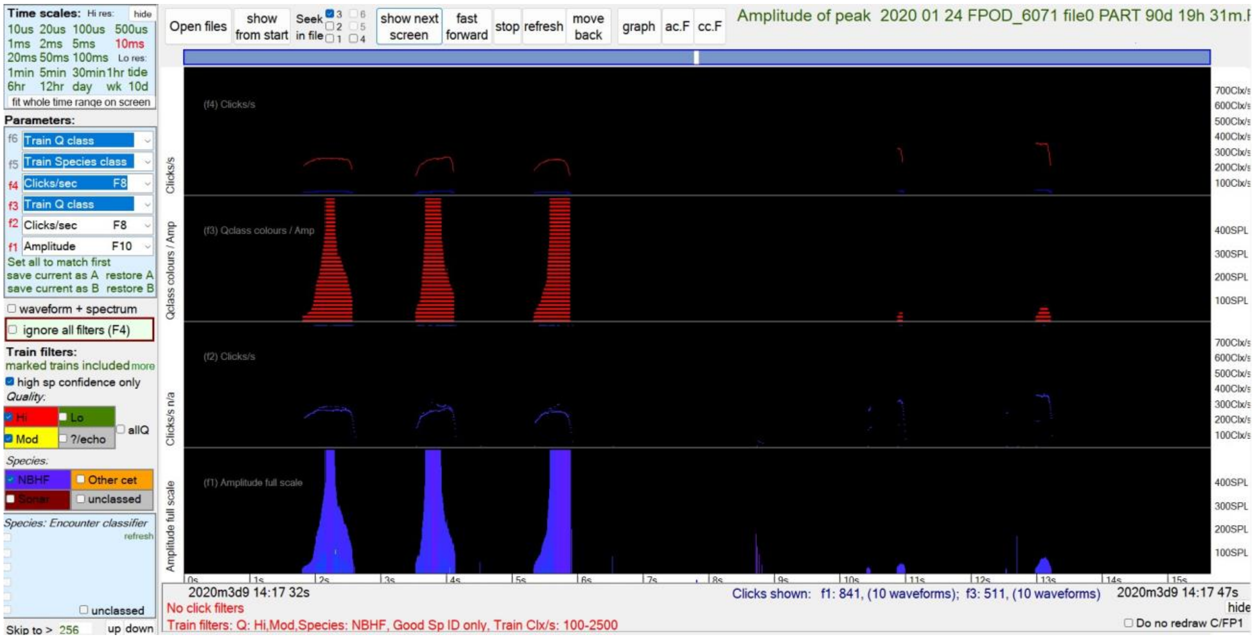

3.

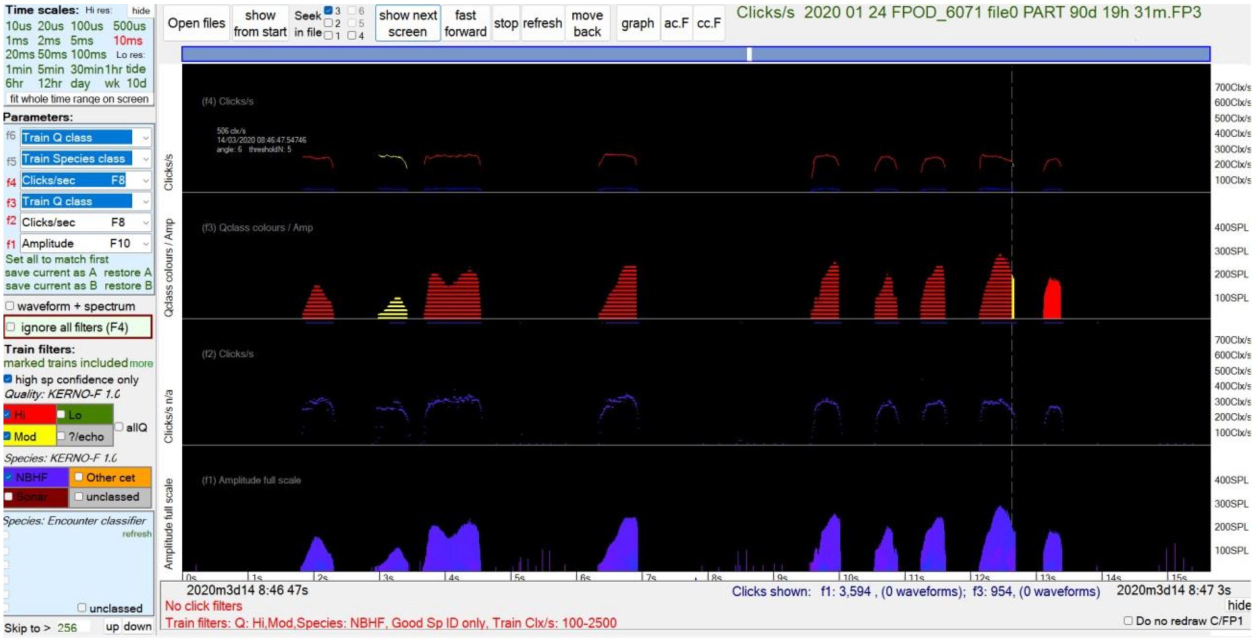
Time scales: Hi res: hide
10us 20us 100us 500us
1ms 2ms 5ms 10ms
20ms 50ms 100ms Lo res:
1min 5min 30min 1hr tide
6hr 12hr day wk 10d
fit whole time range on screen
Parameters:
f6 Train Q class
f5 Train Species class
f4 Clicks/sec F8
f3 Train Q class
f2 Clicks/sec F8
f1 Amplitude F10
Set all to match first
save current as A restore A
save current as B restore B
waveform + spectrum
ignore all filters (F4)
Train filters:
marked trains included more
high sp confidence only
Quality: KERNO-F 1.0
Hi
Lo
Mod
?/echo
allQ
Species: KERNO-F 1.0
NBHF
Other cet
Sonar
unclassed
Species: Encounter classifier
refresh
unclassed
Skip to > 256 up down
Open files
show from start
Seek in file
show next screen
fast forward
stop
refresh
move back
graph
ac.F
cc.F
Clicks/s 2020 01 24 FPOD_6071 file0 PART 90d 19h 31m.FP3
(f4) Clicks/s
(f3) Qclass colours / Amp
(f2) Clicks/s
(f1) Amplitude full scale
Clicks/s
Qclass colours / Amp
Clicks/s n/a
Amplitude full scale
700Clx/s
600Clx/s
500Clx/s
400Clx/s
300Clx/s
200Clx/s
100Clx/s
400SPL
300SPL
200SPL
100SPL
2020m3d14 8:46 47s
Clicks shown: f1: 3,594 , (0 waveforms); f3: 954, (0 waveforms)
2020m3d14 8:47 3s
hide
No click filters
Train filters: Q: Hi,Mod,Species: NBHF, Good Sp ID only, Train Clx/s: 100-2500
Do no redraw C/FP1

4.

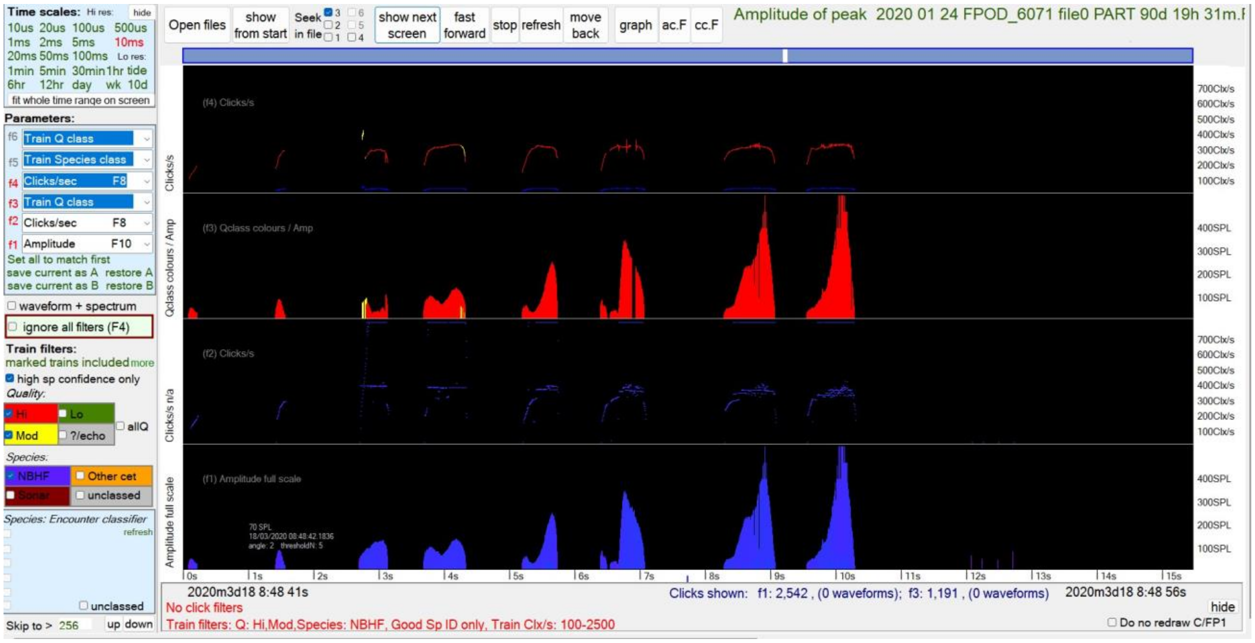
Time scales: Hi res: hide
10us 20us 100us 500us
1ms 2ms 5ms 10ms
20ms 50ms 100ms Lo res:
1min 5min 30min 1hr tide
6hr 12hr day wk 10d
fit whole time range on screen
Parameters:
f6 Train Q class
f5 Train Species class
f4 Clicks/sec F8
f3 Train Q class
f2 Clicks/sec F8
f1 Amplitude F10
Set all to match first
save current as A restore A
save current as B restore B
waveform + spectrum
ignore all filters (F4)
Train filters:
marked trains included more
high sp confidence only
Quality:
Hi
Lo
Mod
?/echo
allQ
Species:
NBHF
Other cet
Sonar
unclassed
Species: Encounter classifier
refresh
unclassed
Skip to > 256 up down
Open files
show from start
Seek in file
show next screen
fast forward
stop
refresh
move back
graph
ac.F
cc.F
Amplitude of peak 2020 01 24 FPOD_6071 file0 PART 90d 19h 31m.F
(f4) Clicks/s
(f3) Qclass colours / Amp
(f2) Clicks/s
(f1) Amplitude full scale
Clicks/s
Qclass colours / Amp
Clicks/s n/a
Amplitude full scale
700Clx/s
600Clx/s
500Clx/s
400Clx/s
300Clx/s
200Clx/s
100Clx/s
400SPL
300SPL
200SPL
100SPL
2020m3d18 8:48 41s
Clicks shown: f1: 2,542 , (0 waveforms); f3: 1,191 , (0 waveforms)
2020m3d18 8:48 56s
hide
No click filters
Train filters: Q: Hi,Mod,Species: NBHF, Good Sp ID only, Train Clx/s: 100-2500
Do no redraw C/FP1

5.

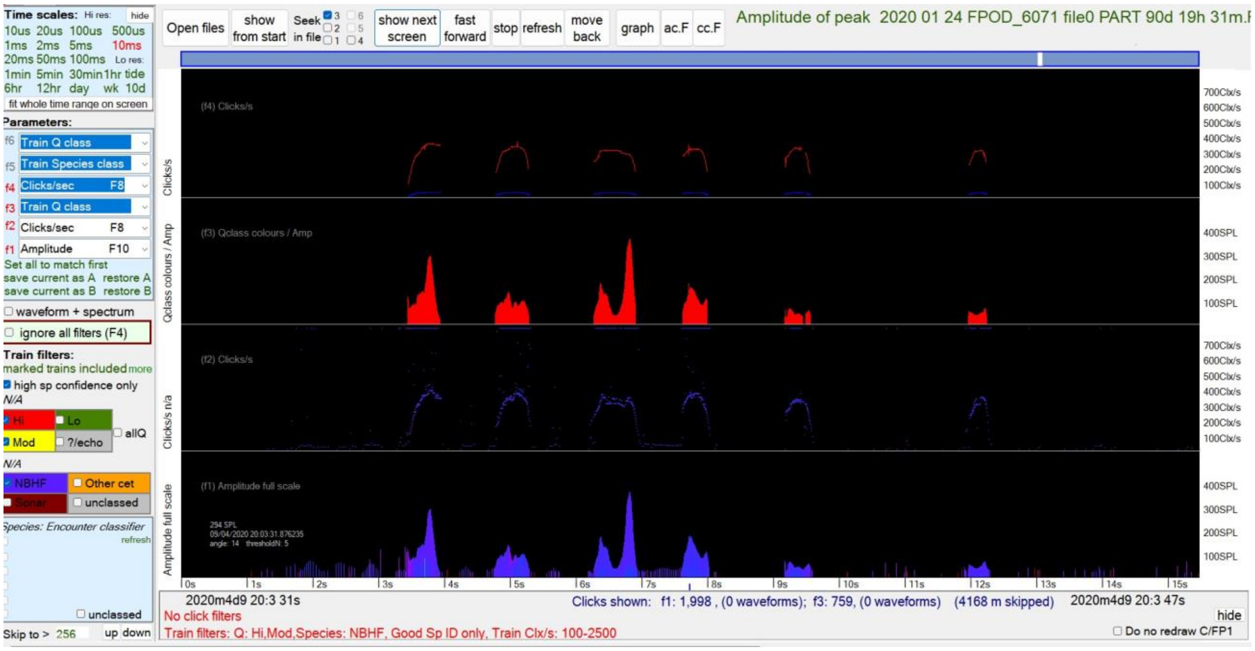

Amplitude of peak 2020 01 24 FPOD_6071 file0 PART 90d 19h 31m.F
Open files
show from start
Seek in file
show next screen
fast forward
stop
refresh
move back
graph
ac.F
cc.F
Time scales:
10us 20us 100us 500us
1ms 2ms 5ms 10ms
20ms 50ms 100ms
1min 5min 30min 1hr tide
6hr 12hr day wk 10d
fit whole time range on screen
Parameters:
Train Q class
Train Species class
Clicks/sec F8
Train Q class
Clicks/sec F8
Amplitude F10
Set all to match first
save current as A restore A
save current as B restore B
waveform + spectrum
ignore all filters (F4)
Train filters:
marked trains included more
high sp confidence only
N/A
Hi
Lo
Mod
?/echo
allQ
N/A
NBHF
Other cet
unclassed
Species: Encounter classifier
refresh
unclassed
Skip to > 256 up down
(f4) Clicks/s
(f3) Qclass colours / Amp
(f2) Clicks/s
(f1) Amplitude full scale
700Clx/s
600Clx/s
500Clx/s
400Clx/s
300Clx/s
200Clx/s
100Clx/s
400SPL
300SPL
200SPL
100SPL
0s 1s 2s 3s 4s 5s 6s 7s 8s 9s 10s 11s 12s 13s 14s 15s
2020m4d9 20:3 31s
Clicks shown: f1: 1,998 , (0 waveforms); f3: 759, (0 waveforms) (4168 m skipped)
2020m4d9 20:3 47s
hide
No click filters
Train filters: Q: Hi,Mod,Species: NBHF, Good Sp ID only, Train Clx/s: 100-2500
Do no redraw C/FP1

6.

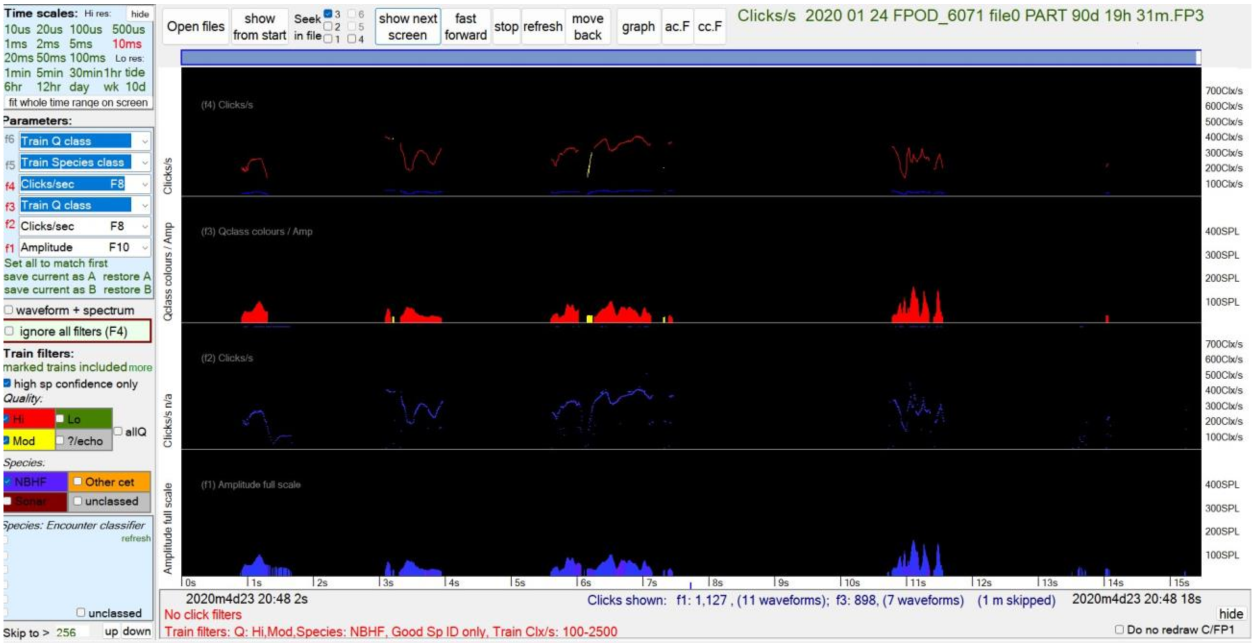

Clicks/s 2020 01 24 FPOD_6071 file0 PART 90d 19h 31m.FP3
Open files
show from start
Seek in file
show next screen
fast forward
stop
refresh
move back
graph
ac.F
cc.F
Time scales:
10us 20us 100us 500us
1ms 2ms 5ms 10ms
20ms 50ms 100ms
1min 5min 30min 1hr tide
6hr 12hr day wk 10d
fit whole time range on screen
Parameters:
Train Q class
Train Species class
Clicks/sec F8
Train Q class
Clicks/sec F8
Amplitude F10
Set all to match first
save current as A restore A
save current as B restore B
waveform + spectrum
ignore all filters (F4)
Train filters:
marked trains included more
high sp confidence only
Quality:
Hi
Lo
Mod
?/echo
allQ
Species:
NBHF
Other cet
unclassed
Species: Encounter classifier
refresh
unclassed
Skip to > 256 up down
(f4) Clicks/s
(f3) Qclass colours / Amp
(f2) Clicks/s
(f1) Amplitude full scale
700Clx/s
600Clx/s
500Clx/s
400Clx/s
300Clx/s
200Clx/s
100Clx/s
400SPL
300SPL
200SPL
100SPL
0s 1s 2s 3s 4s 5s 6s 7s 8s 9s 10s 11s 12s 13s 14s 15s
2020m4d23 20:48 2s
Clicks shown: f1: 1,127 , (11 waveforms); f3: 898, (7 waveforms) (1 m skipped)
2020m4d23 20:48 18s
hide
No click filters
Train filters: Q: Hi,Mod,Species: NBHF, Good Sp ID only, Train Clx/s: 100-2500
Do no redraw C/FP1

7.

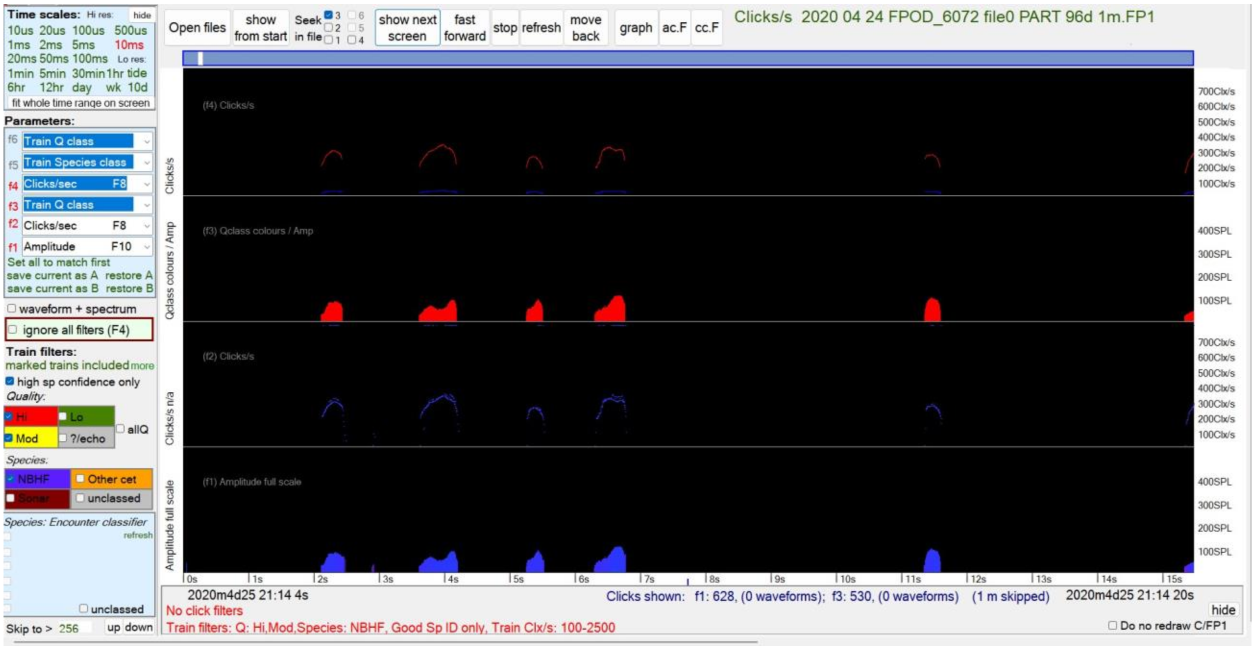
Time scales: Hi res: hide
10us 20us 100us 500us
1ms 2ms 5ms 10ms
20ms 50ms 100ms Lo res:
1min 5min 30min 1hr tide
6hr 12hr day wk 10d
fit whole time range on screen
Parameters:
f6 Train Q class
f5 Train Species class
f4 Clicks/sec F8
f3 Train Q class
f2 Clicks/sec F8
f1 Amplitude F10
Set all to match first
save current as A restore A
save current as B restore B
waveform + spectrum
ignore all filters (F4)
Train filters:
marked trains included more
high sp confidence only
Quality:
Hi Lo
Mod ?/echo allQ
Species:
NBHF Other cet
Sonar unclassed
Species: Encounter classifier
refresh
unclassed
Skip to > 256 up down
Open files
show from start
Seek in file 3 2 1 6 5 4
show next screen
fast forward
stop refresh
move back
graph ac.F cc.F
Clicks/s 2020 04 24 FPOD_6072 file0 PART 96d 1m.FP1
(f4) Clicks/s
Clicks/s
700Clx/s 600Clx/s 500Clx/s 400Clx/s 300Clx/s 200Clx/s 100Clx/s
(f3) Qclass colours / Amp
Qclass colours / Amp
400SPL 300SPL 200SPL 100SPL
(f2) Clicks/s
Clicks/s n/a
700Clx/s 600Clx/s 500Clx/s 400Clx/s 300Clx/s 200Clx/s 100Clx/s
(f1) Amplitude full scale
Amplitude full scale
400SPL 300SPL 200SPL 100SPL
0s 1s 2s 3s 4s 5s 6s 7s 8s 9s 10s 11s 12s 13s 14s 15s
2020m4d25 21:14 4s
Clicks shown: f1: 628, (0 waveforms); f3: 530, (0 waveforms) (1 m skipped)
2020m4d25 21:14 20s
hide
No click filters
Train filters: Q: Hi,Mod,Species: NBHF, Good Sp ID only, Train Clx/s: 100-2500
Do no redraw C/FP1

8.

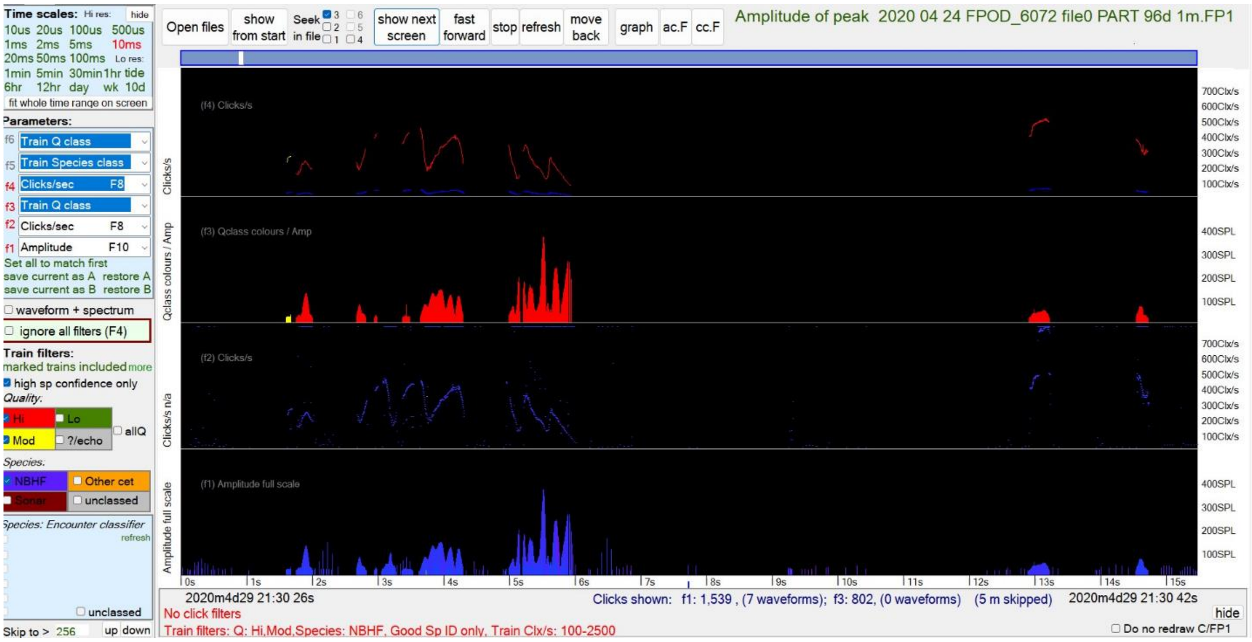
Time scales: Hi res: hide
10us 20us 100us 500us
1ms 2ms 5ms 10ms
20ms 50ms 100ms Lo res:
1min 5min 30min 1hr tide
6hr 12hr day wk 10d
fit whole time range on screen
Parameters:
f6 Train Q class
f5 Train Species class
f4 Clicks/sec F8
f3 Train Q class
f2 Clicks/sec F8
f1 Amplitude F10
Set all to match first
save current as A restore A
save current as B restore B
waveform + spectrum
ignore all filters (F4)
Train filters:
marked trains included more
high sp confidence only
Quality:
Hi Lo
Mod ?/echo allQ
Species:
NBHF Other cet
Sonar unclassed
Species: Encounter classifier
refresh
unclassed
Skip to > 256 up down
Open files
show from start
Seek in file 3 2 1 6 5 4
show next screen
fast forward
stop refresh
move back
graph ac.F cc.F
Amplitude of peak 2020 04 24 FPOD_6072 file0 PART 96d 1m.FP1
(f4) Clicks/s
Clicks/s
700Clx/s 600Clx/s 500Clx/s 400Clx/s 300Clx/s 200Clx/s 100Clx/s
(f3) Qclass colours / Amp
Qclass colours / Amp
400SPL 300SPL 200SPL 100SPL
(f2) Clicks/s
Clicks/s n/a
700Clx/s 600Clx/s 500Clx/s 400Clx/s 300Clx/s 200Clx/s 100Clx/s
(f1) Amplitude full scale
Amplitude full scale
400SPL 300SPL 200SPL 100SPL
0s 1s 2s 3s 4s 5s 6s 7s 8s 9s 10s 11s 12s 13s 14s 15s
2020m4d29 21:30 26s
Clicks shown: f1: 1,539 , (7 waveforms); f3: 802, (0 waveforms) (5 m skipped)
2020m4d29 21:30 42s
hide
No click filters
Train filters: Q: Hi,Mod,Species: NBHF, Good Sp ID only, Train Clx/s: 100-2500
Do no redraw C/FP1
