# Supplementary material for: Reading between the lines: Novel insights on wild Pacific harbour porpoise (Phocoena phocoena vomerina) social communication through narrow-band high frequency click trains
Source: PLoS One. 2025 Feb 12;20(2):e0317727. doi: 10.1371/journal.pone.0317727 (PMC11819512; doi:10.1371/journal.pone.0317727)
Supplement: S1 Table — (DOCX) [file pone.0317727.s002.docx]

S1 Table. Summary of detection positive minutes (DPM) and total F-POD recorded minutes at Tuck Islet between 2020-2022.

| **Year** | **Month** | **Social DPM** | **Total DPM** | **Total Minutes Recorded** |
| --- | --- | --- | --- | --- |
| 2020 | Jan | 2 | 31 | 10620 |
|  | Feb | 10 | 215 | 41760 |
|  | Mar | 28 | 449 | 44640 |
|  | Apr | 120 | 1146 | 42960 |
|  | May | 776 | 4799 | 44640 |
|  | Jun | 135 | 2313 | 43200 |
|  | Jul | 64 | 1382 | 44640 |
|  | Aug | 27 | 418 | 44640 |
|  | Sep | 13 | 327 | 43200 |
|  | Oct | 24 | 694 | 44400 |
|  | Nov | 142 | 2051 | 43200 |
|  | Dec | 48 | 1181 | 44640 |
| **2020 Total** |  | **1389** | **15006** | **492540** |
| 2021 | Jan | 35 | 1054 | 44640 |
|  | Feb | 12 | 455 | 40320 |
|  | Mar | 43 | 740 | 44640 |
|  | Apr | 72 | 1345 | 43200 |
|  | May | 109 | 2082 | 44640 |
|  | Jun | 47 | 1324 | 43200 |
|  | Jul | 43 | 737 | 44280 |
|  | Aug | 11 | 440 | 44640 |
|  | Sep | 2 | 281 | 43200 |
|  | Oct | 23 | 476 | 40260 |
|  | Nov | 4 | 188 | 43200 |
|  | Dec | 15 | 428 | 44640 |
| **2021 Total** |  | **416** | **9550** | **520860** |
| 2022 | Jan | 6 | 193 | 42960 |
|  | Feb | 2 | 41 | 40320 |
|  | Mar | 15 | 360 | 44640 |
|  | Apr | 7 | 576 | 43200 |
|  | May | 61 | 1615 | 44460 |
|  | Jun | 144 | 1808 | 43200 |
|  | Jul | 34 | 1030 | 44640 |
|  | Aug | 11 | 485 | 44640 |
|  | Sep | 18 | 443 | 43200 |
|  | Oct | 2 | 89 | 26280 |
|  | Nov | 2 | 106 | 43200 |
|  | Dec | 3 | 202 | 44640 |
| **2022 Total** |  | **305** | **6948** | **505380** |
| **Grand Total** |  | **2110** | **31504** | **1518780** |
