# Supplementary figures and images for: Reading between the lines: Novel insights on wild Pacific harbour porpoise (Phocoena phocoena vomerina) social communication through narrow-band high frequency click trains
Source: PLoS One. 2025 Feb 12;20(2):e0317727. doi: 10.1371/journal.pone.0317727 (PMC11819512; doi:10.1371/journal.pone.0317727)

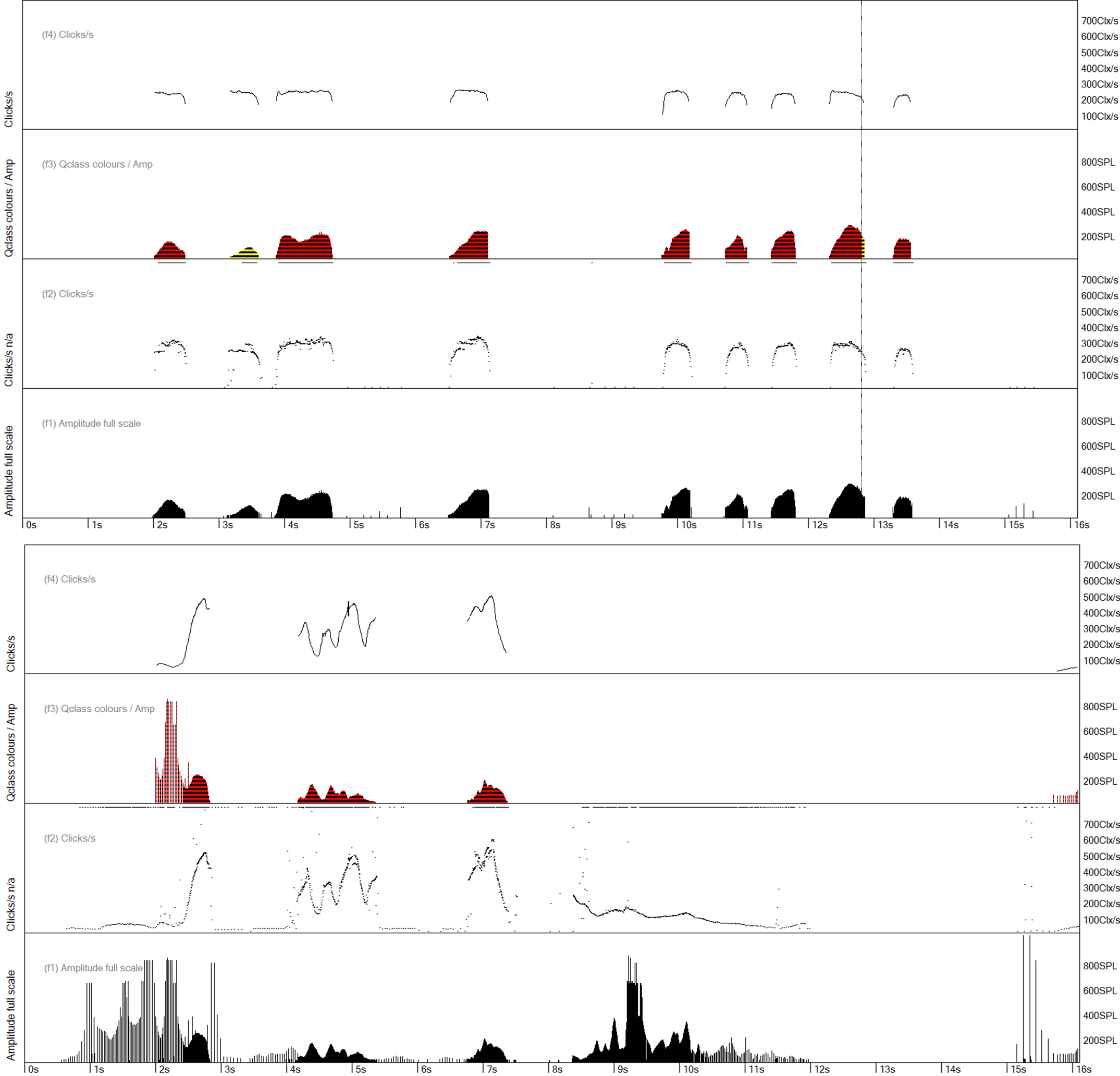

Supplement: S1 Fig — (TIF) [file pone.0317727.s003.tif]

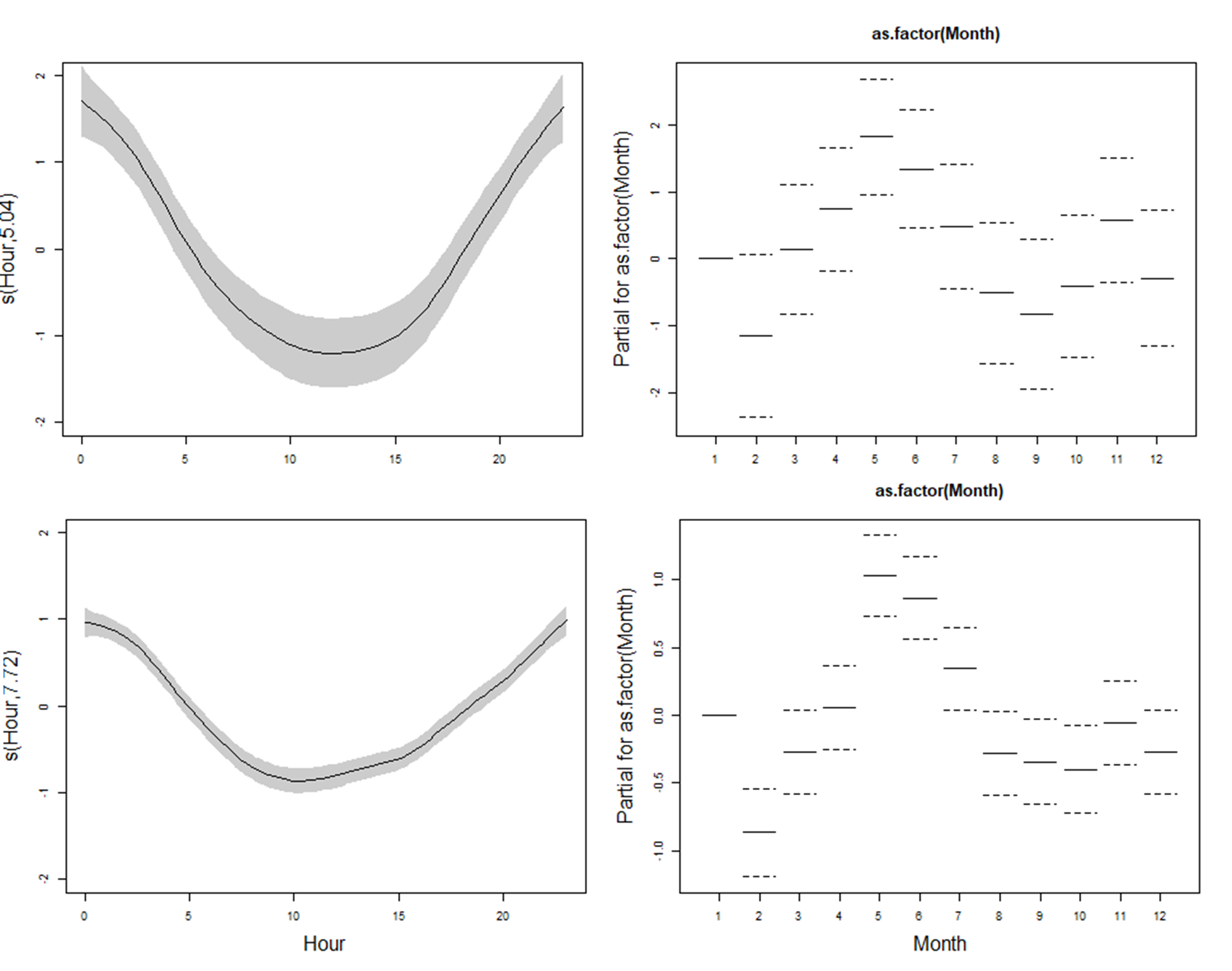

Supplement: S2 Fig — (TIF) [file pone.0317727.s004.tif]

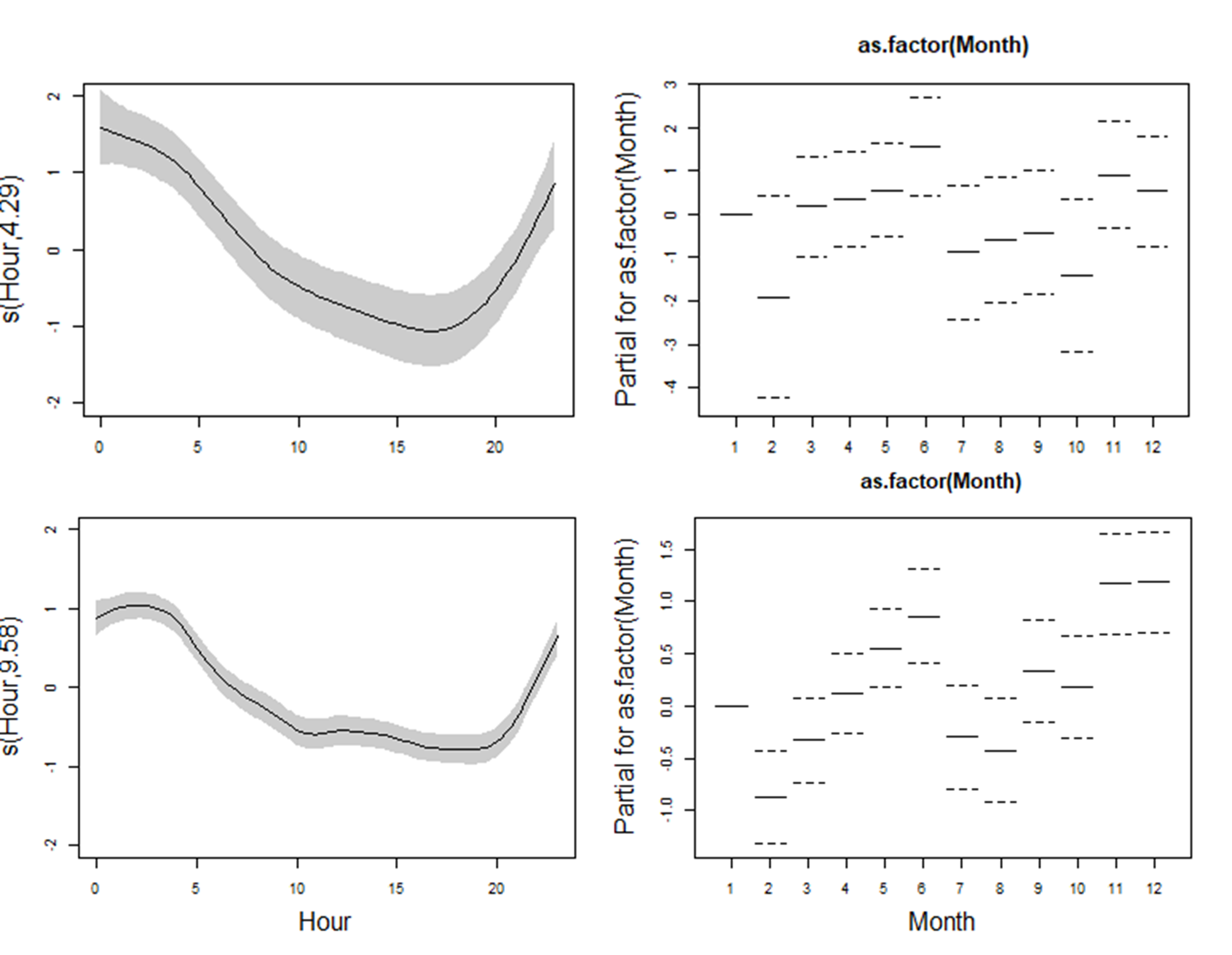

Supplement: S3 Fig — (TIF) [file pone.0317727.s005.tif]
